# Fungal Kinases With a Sweet Tooth: Pleiotropic Roles of Their Phosphorylated Inositol Sugar Products in the Pathogenicity of *Cryptococcus neoformans* Present Novel Drug Targeting Opportunities

**DOI:** 10.3389/fcimb.2019.00248

**Published:** 2019-07-15

**Authors:** Sophie Lev, Cecilia Li, Desmarini Desmarini, Tania C. Sorrell, Adolfo Saiardi, Julianne T. Djordjevic

**Affiliations:** ^1^Centre for Infectious Diseases and Microbiology, The Westmead Institute for Medical Research, Sydney, NSW, Australia; ^2^Sydney Medical School-Westmead, The University of Sydney, Sydney, NSW, Australia; ^3^Marie Bashir Institute for Infectious Diseases and Biosecurity, University of Sydney, Sydney, NSW, Australia; ^4^Centre for Infectious Diseases and Microbiology–Public Health, NSW Health Pathology, Westmead Hospital, Sydney, NSW, Australia; ^5^MRC Laboratory for Molecular Cell Biology, University College London, London, United Kingdom

**Keywords:** inositol polyphosphate kinase, inositol pyrophosphate, IP_7_, IP, PP-IP, virulence, signaling, *Cryptococcus neoformans*

## Abstract

Invasive fungal pathogens cause more than 300 million serious human infections and 1.6 million deaths per year. A clearer understanding of the mechanisms by which these fungi cause disease is needed to identify novel targets for urgently needed therapies. Kinases are key components of the signaling and metabolic circuitry of eukaryotic cells, which include fungi, and kinase inhibition is currently being exploited for the treatment of human diseases. Inhibiting evolutionarily divergent kinases in fungal pathogens is a promising avenue for antifungal drug development. One such group of kinases is the phospholipase C1-dependent inositol polyphosphate kinases (IPKs), which act sequentially to transfer a phosphoryl group to a pre-phosphorylated inositol sugar (IP). This review focuses on the roles of fungal IPKs and their IP products in fungal pathogenicity, as determined predominantly from studies performed in the model fungal pathogen *Cryptococcus neoformans*, and compares them to what is known in non-pathogenic model fungi and mammalian cells to highlight potential drug targeting opportunities.

Invasive fungal diseases threaten human and animal health and food security. Invasive fungal diseases have caused recent major dieoffs and extinctions in wild animal species and plants, forcing overuse of antifungals to sustain our food supply (Fisher et al., 2012; Editorial Stop neglecting fungi, 2017). World-wide, invasive fungal pathogens cause more than 300 million serious human infections and 1.6 million deaths per year (Editorial Stop neglecting fungi, 2017). Infections caused by *Cryptococcus neoformans, Candida albicans* and *Aspergillus fumigatus* are common in immunocompromised individuals (e.g., those with HIV, blood cancers, and organ transplants) and a lack of effective antifungal drugs has compromised recent medical advances in the treatment of these conditions. Current antifungal agents are either toxic, lack broad-spectrum activity or are sub-optimally efficacious due to poor absorption, contributing to side effects and high rates of morbidity and mortality (Brown et al., 2012). Prolonged treatment courses breed drug resistance. Despite the urgent need, no new drug classes have been marketed since the introduction of echinocandins in 2002 (Denning, 2002).

Kinases are key components of signaling and metabolic pathways in all eukaryotes, including fungi. *In silico* predictions in *C. neoformans* identified 183 kinases, and experimental data show that 63 of them play a role in pathogenicity (Lee et al., 2016). The inhibition of kinases by small molecules is of major interest to pharmaceutical companies. With over 30 kinase inhibitors approved for treating non-infectious diseases, selective inhibition of fungal kinase activity is a potentially appealing antifungal therapeutic strategy. Kinases catalyze the transfer of phosphoryl groups from high energy molecules, predominantly ATP, to a variety of substrates. Eukaryotic protein kinases (where the substrate is a protein) are the most-well characterized. In contrast, kinases that target lipids and phosphorylated sugars are less well-studied.

Despite their reduced prevalence, these latter kinases provide a source of secondary messengers, which have crucial roles in signal transduction. Inhibitors targeting phosphoinositide kinases, which use the lipid phosphatidylinositol or its phosphorylated derivatives (e.g., PIP_2_) as substrates are under investigation as anticancer agents (Fabbro, 2015). They transfer a phosphoryl group to a specific position on the inositol ring of the phospholipid head group. In contrast, the inositol polyphosphate kinases (IPKs), which are often confused with the phosphoinositide kinases, are distinct in that they phosphorylate a variety of cytosolic, water-soluble inositol polyphosphates (IPs) originating from the phosphatidylinositol head group. In yeast, the first of these substrates is inositol trisphosphate (IP_3_). IP_3_ is generated by the hydrolytic action of the delta isoform of PLC (PLC1 in yeast) on PIP_2_. IPKs sequentially phosphorylate IP_3_ to create a combination of molecules with monophosphates at various positions on the inositol ring (IPs), or a combination of monophosphates and di- (or pyro-) phosphates (PP-IPs). The PP moiety harbors a “high-energy” phosphoanhydride bond, similar to ATP. Presumably due to their function as phosphate donors, PP-IPs undergo rapid turnover in eukaryotic cells (Menniti et al., 1993). Both the phosphorylated lipid products of phosphoinositide kinases and the soluble IPs/PP-IPs products of IPKs regulate diverse cellular processes in eukaryotes, including proliferation, survival, cytoskeletal arrangement, vesicle traffic, glucose transport, and platelet function (Fruman et al., 1998; Sarmah and Wente, 2010). Perturbed synthesis of IPs is also linked to apoptosis and insulin secretion (Nagata et al., 2005; Illies et al., 2007; Sarmah and Wente, 2010), and developmental defects in vertebrates (Frederick et al., 2005; Sarmah et al., 2005). IPs also regulate DNA repair, chromatin remodeling, transcription and gene expression, mRNA export, telomere length, exocytosis, RNA editing, translation, and Ca^2+^ channels (York et al., 1999; Shen et al., 2003; Steger et al., 2003; Macbeth et al., 2005; Saiardi et al., 2005; Alcazar-Roman and Wente, 2008; Bolger et al., 2008; Sarmah and Wente, 2009).

## Identification of the PLC1-dependent IPK Pathway as a New Virulence-Related Signaling Pathway in the Model Fungal Pathogen *C. neoformans*

The IPK pathway has been characterized in mammalian cells and in model (non-pathogenic) yeast. The most substantial progress in characterizing the pathway in a fungal pathogen has come from studies performed in *C. neoformans* (Lev et al., 2013, 2015; Li et al., 2016; Li C. et al., 2017). To date, the only non-fungal pathogen in which kinases in the pathway have been studied is the protozoan parasite, *Trypanosoma brucei*, which causes sleeping sickness (Cordeiro et al., 2017).

**Figure 1:**
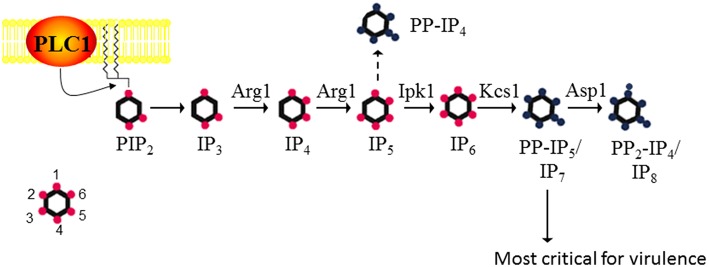
The inositol polyphosphate biosynthesis pathway in *C. neoformans*. PLC1 –derived IP_3_ is sequentially phosphorylated to IP_4_, IP_5_, and IP_6_ by Arg1 and Ipk1. Kcs1 generates PP-IP_4_ and PP-IP_5_/IP_7_ from IP_5_ and IP_6_, respectively. However, PP-IP_4_ is only detected in the absence of Ipk1 (hence the dashed line). PP_2_-IP_4_ is derived from Asp1. The insert represents the position of the phosphates on the inositol ring. Figure was adapted from Lev et al. (2015), Li et al. (2016).

*C. neoformans* is a respiratory pathogen that disseminates to the central nervous system. It causes fatal meningitis in about a quarter of a million people per year. The majority of these fatalities occur in individuals living in Sub-Saharan Africa and South East Asia, who have limited access to appropriate antifungal drugs (Rajasingham et al., 2017). The advantages of using *C. neoformans* as a working model are its genetically tractable and annotated haploid genome, and the availability of robust vertebrate and invertebrate infection models. Using *S. cerevisiae* IPK protein sequences in homology searches, we identified a series of putative IPK-encoding genes in the *C. neoformans* strain H99 genome and designated them Arg1, Arg2, Ipk1, Kcs1, and Vip1/Asp1 (Figure 1). Similar to the fission yeast, *Schizosaccharomyces pombe*, Vip1 is referred to as Asp1 in the cryptococcal database (Topolski et al., 2016; Pascual-Ortiz et al., 2018). The most striking difference between the yeast and mammalian IPK pathways is the linearity, and hence lack of redundancy in the yeast pathway. In mammalian cells, there is substantial redundancy, with 3 alternative enzymes able to convert IP_3_ to IP_5_ (Li et al., 2016).

Metabolic profiling of cryptococcal (Cn) IPK deletion mutants was used to determine the substrate specificity of each IPK. CnArg1 and CnArg2 are most similar to the *S. cerevisiae* (Sc) IP_3_/IP_4_ kinase, ScArg82/Ipk2/ArgIII/IPMK (Lev et al., 2013; Li C. et al., 2017). However, only CnArg1 plays a role in IP metabolism in *C. neoformans*: in the *ARG1* deletion mutant (*arg1*Δ), but not the *arg2*Δ mutant, soluble IP_3_ accumulated and was not further phosphorylated to IP_4−8_ (Lev et al., 2015). Furthermore, enzymatic assays using recombinant CnArg1 confirmed that this kinase uses ATP to convert IP_3_ to IP_4_ and IP_5_ (Li C. et al., 2017), indicating that CnArg1 is the major IP_3_ kinase in *C. neoformans*. It was shown that the I(1,4,5)P_3_ substrate of Arg1 is derived from phosphatidylinositol 4,5-bisphosphate (PIP_2_) via the hydrolytic activity of phospholipase C1 (PLC1) (Chayakulkeeree et al., 2008; Lev et al., 2013). CnArg1 could restore defective phenotypes in the Sc*arg82*Δ mutant, confirming that CnArg1 is the ortholog of ScArg82 (Li C. et al., 2017). CnArg2 has no identified cellular function but is unlikely to be a pseudo-kinase as it retains the conserved “P-x-x-x-D-x-K-x-G” catalytic motif essential for IP_3_ kinase activity in ScArg82 and CnArg1 (Saiardi et al., 1999; Bertsch et al., 2000). The reason for IP_3_ kinase gene duplication in this basidiomycetous yeast but not in the ascomycetes is unknown, but may reflect evolutionary diversification.

Metabolic profiling confirmed that CnIpk1 is the major IP_5_ kinase in *C. neoformans*, using IP_5_ as a substrate to produce a molecule with a fully phosphorylated inositol ring, namely, IP_6_ (Li et al., 2016). CnKcs1 and CnAsp1 were shown to be inositol pyrophosphate synthases: CnKcs1 phosphorylates IP_6_ at position 5 generating 5-PP-IP_5_ (IP_7_), which is further phosphorylated by CnAsp1 at position 1 to produce 1,5-PP_2_-IP_4_ (IP_8_)(Lev et al., 2015). In *S. cerevisiae*, the homolog of Asp1, Vip1, also phosphorylates IP_6_, producing 1-PP-IP_5_. However, this ScVip1 activity is very low as the 1-PP-IP_5_ product can only be detected by HPLC when both Sc*KCS1* and the inositol pyrophosphatase-encoding gene, Sc*DDP1*, are deleted, the assumption being that any 1-PP-IP_5_ made by Asp1 is broken down by ScDdp1 (Mulugu et al., 2007). Whether Asp1 can phosphorylate IP_6_ in *C. neoformans*, to produce 1-PP-IP_5_, remains to be determined.

Cn*arg1*Δ, Cn*kcs1*Δ, and Cn*ipk1*Δ were either avirulent or severely attenuated in virulence in a mouse inhalation model as discussed in the next section (Lev et al., 2013, 2015; Li et al., 2016; Li C. et al., 2017). This was in contrast to Cn*arg2*Δ and Cn*asp1*Δ, which retained a WT-like virulence profile. The importance of CnArg1 and CnKcs1 in cryptococcal cellular function was also highlighted by the fact that neither deletion mutant is represented in a signature-tagged kinome mutant library created by Lee et al in a genome-wide study of cryptococcal kinases (Lee et al., 2016). Only the Cn*ipk1*Δ and Cn*asp1*Δ mutants are present in this library, with CnIpk1 also implicated in pathogenicity (Lee et al., 2016).

## IP_3_ Kinase, Arg1, has the Most Significant Impact on Fungal Cellular Function and Pathogenicity, Acting via its IP Products

**Figure 2:**
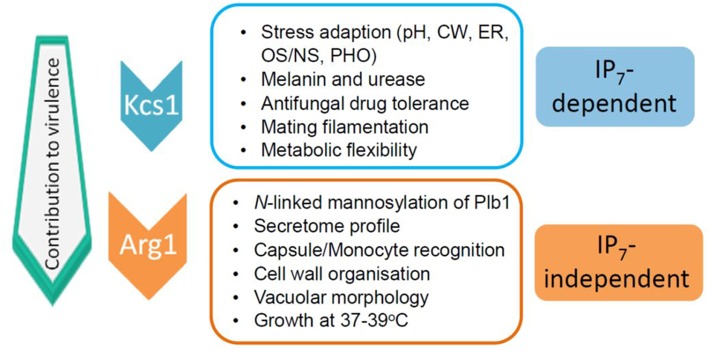
IP_7_-dependent and IP_7_-independent functions conveyed via CnArg1. Due to these combined functions, the contribution of CnArg1 to fungal virulence is greater than that of CnKcs1. pH—alkaline pH stress; CW—cell wall stress induced by calcofluor white, Congo red, SDS and caffeine; ER—endoplasmic reticulum stress caused by tunicamycin and DTT; OS/NS—oxidative and nitrosative stress; PHO—low phosphate.

The impact of the loss of IP_3_ kinase function has been studied in *C. neoformans* and *C. albicans*. Compared to other IPKs, loss of CnArg1 had the most debilitating effect on cryptococcal phenotype (Li C. et al., 2017), while the phenotype and metabolic profile of the Cn*arg2*Δ mutant was similar to that of WT (Lev et al., 2013). Cn*arg1*Δ phenotypes included a significant reduction in growth at human body temperature (37°C) in either rich (YPD) or a more physiological cell culture medium (RPMI/5%CO_2_), a cell separation defect and enlarged vacuoles (Lev et al., 2013; Li C. et al., 2017). Additional defects included reduced mating filamentation and production of the virulence factors urease and melanin, and a cell wall integrity defect (Lev et al., 2013; Li C. et al., 2017). The latter was associated with a thickened cell wall and hypermannosylation of the cell wall-associated/secreted virulence determinant phospholipase B1 (PLB1) (Li C. et al., 2017). Although PLB1 release into the culture medium was attenuated, more cell-associated PLB1 was consistent with a secretion block. Capsules of the Cn*arg1*Δ mutant were smaller than those of WT, and this defect correlated with a higher rate of Cn*arg1*Δ phagocytosis by human peripheral blood monocytes and rapid clearance from lung in a mouse infection model (Li C. et al., 2017). The Cn*arg1*Δ mutant also exhibited reduced growth in the presence of non-glucose carbon sources and during phosphate starvation (Li C. et al., 2017). Cn*arg1*Δ phenotypes are summarized in Figure 2.

In a recent study by Li J. et al. (2017), the impact of loss of the IP_3_ kinase gene (referred to as *IPK2*) was investigated in diploid *C. albicans*. The authors were unable to delete both copies of Ca*IPK2*, consistent with an essential role for Ca*IPK2* in cellular function. Instead they created a heterozygous *IPK2* deletion mutant and controlled expression of the remaining *IPK2* allele using the inducible MET3 promoter. In contrast to the suppression of virulence-associated functions observed in *C. neoformans* following deletion of *ARG1*, suppression of Ca*IPK2* in *C. albicans* promoted virulence-related functions, such as hyphal development, by increasing expression of hyphal-specific genes and the transport of hypha-specific factors, and secretion of degradative enzymes such as proteases and lipases, which would include phospholipases (Li J. et al., 2017). Hyphal development and secretion of degradative enzymes allow *C. albicans* to invade the epithelial barrier and avoid detection by the innate immune response. The effect of diminished *IPK2* gene expression on the virulence of *C. albicans* in animal infection models remains to be determined.

Although *C. neoformans* does not form hyphae during vegetative growth, it does alter its morphology during mating. However, loss of CnArg1 reduced mating-associated filamentation in *C. neoformans* (Lev et al., 2013). The functional significance of the differing roles of CnArg1 and CaIpk2 in regulating morphology in these two fungal pathogens remains to be determined. In contrast, overproduction of lipases, in particular PLB1, was a consistent phenotype in Arg1-deficient *C. neoformans* and *C. albicans*. Although increased PLB1 abundance on the cryptococcal cell surface could potentially lead to enhanced virulence, the opposite was found to be true (Li C. et al., 2017). Li J. et al. (2017) also found that loss of IPK in *C. albicans* increased damage to macrophages. Although macrophage damage by Cn*arg1*Δ was not assessed, increased uptake of the Cn*arg1*Δ mutant by blood monocytes was observed (Li C. et al., 2017). This may have resulted in a higher rate of macrophage death due to the oxidative burst directed at the internalized pathogen. Similar to the mammalian and *S. cerevisiae* Ipk2/Arg82 homologs, CaIpk2 was observed to be nuclear (Li J. et al., 2017). The cellular location of CnArg1 remains to be determined.

### Potential Lipid Substrates of IP_3_ Kinases

Another potential metabolic function of Arg1 is to phosphorylate PIP_2_ and thus act as a PI3K. Both human and yeast IPMKs (i.e. *ARG82/IPK2*) display PI3K activity *in vitro* (Resnick et al., 2005). Using mammalian cell transfection studies, Resnick et al. (2005) showed that human IPMKs were exclusively localized in the nucleus and exhibited wortmannin-insensitive PI3K activity to regulate transcription. In contrast, canonical PI3Ks are predominantly cytosolic and wortmannin-sensitive. The importance of Arg82 PI3K activity *in vivo* has not been directly addressed. The cellular location of CnArg1 and whether CnArg1 functions as a PI3K to use membrane phosphoinositides as a substrate, also remains to be determined.

### Functions of IP_3_ Kinases Which Are Independent of Catalytic Activity

In *S. cerevisiae*, Arg82 is nuclear-localized and possesses functions independent of its catalytic activity. Using a kinase-dead mutant strain in which key catalytic residues were mutated, ScArg82 was shown to be a regulatory component of the arginine-sensitive ArgR-Mcm1 transcriptional complex, which is required for regulating gene expression associated with arginine synthesis and breakdown (Bechet et al., 1970; Dubois et al., 1987; Bosch and Saiardi, 2012). These data led to the assignment of the “Arg” nomenclature. Given that the Arg1 homolog in *C. albicans* is also nuclei-localized, it too could potentially function to regulate gene expression (Li J. et al., 2017). Whether CnArg1 is nuclear and whether all of the attenuated phenotypes associated with the Cn*arg1*Δ mutant are due solely to the absence of one or more of its enzymatic products remains to be determined. However, due to the Cn*arg1*Δ mutant sharing several phenotypic defects with the Cn*kcs1*Δ and the CnKcs1 kinase-dead mutants (Lev et al., 2015), many of the key functions of Arg1 in pathogenicity are attributable to IP_7_ as discussed below.

## Hierarchy of Importance of IPs in Fungal Pathogenesis Favors PP-IP_4_ and PP-IP_5_/IP_7_

### Role of IP_8_

To dissect the importance of cryptococcal IPs downstream of IP_3_ in cellular function, the phenotypes of the WT, Cn*ipk1*Δ, Cn*ipk1*Δ*kcs1*Δ, Cn*kcs1*Δ, and Cn*asp1*Δ deletion mutants, and their virulence profile in animal models, were compared. Taking this approach, the impact of absent metabolites on cellular function could be assessed. The *asp1*Δ mutant was found to accumulate IP_7_ and was deficient in IP_8_. No impact of absent IP_8_ on cellular function was observed as the Cn*asp1*Δ mutant exhibited a WT phenotype under all growth conditions tested. As expected from the phenotypic assessment, Cn*asp1*Δ virulence was similar to WT in a mouse inhalation model (Lev et al., 2015). In contrast, IP_8_ is required for cellular functions such as dimorphic switching, polarized growth and microtubule cytoskeletal regulation in *S. pombe, Aspergillus nidulans* and the basidiomycete, *Ustilago maydis* (Feoktistova et al., 1999; Pohlmann and Fleig, 2010; Pohlmann et al., 2014). In addition to IP_8_, Vip1 in *S. cerevisiae* produces 1-PP-IP_5_. It is interesting to note that the structure of ScVip1 is atypical, and differs significantly from other IPKs: it contains an N-terminal kinase domain and a C-terminal histidine acid phosphatase domain. This acid phosphatase domain functions to repress the activity of the kinase domain (Pascual-Ortiz et al., 2018).

### Role of IP_7_

In contrast to IP_8_, the absence of its precursor, IP_7_ in the Cn*kcs1*Δ mutant had a profound impact on phenotype and virulence. However, the effect was not as marked as that in the Cn*arg1*Δ mutant, which is also deficient in IP_7_. A comparison of the phenotypes of the two mutants allowed IP_7_-dependent and IP_7_-independent functions to be distinguished as summarized in Figure 2 (Lev et al., 2015; Li C. et al., 2017). The Cn*kcs1*Δ mutant had a less severe 37°C growth defect than the Cn*arg1*Δ mutant in rich medium (although the temperature defect for both mutants was similar in the more physiological cell culture medium). The Cn*kcs1*Δ mutant also had a cell wall integrity defect but displayed neither the abnormal multi-layered, thickened cell walls nor the enlarged vacuoles observed for the Cn*arg1*Δ mutant. Cn*arg1*Δ cells were enlarged, while Cn*kcs1*Δ cells were WT-like. The Cn*kcs1*Δ mutant also produced lower amounts of the virulence factors, melanin, urease and PLB1 but had larger and more mucoid capsules compared to the Cn*arg1*Δ mutant. The PLB1 production defect in the *kcs1*Δ mutant was different to that of the Cn*arg1*Δ mutant: relative to WT and the Cn*arg1*Δ mutant, the Cn*kcs1*Δ mutant produced less total PLB1 (cell-associated and secreted), while only the release of cell-associated PLB1 was affected (i.e. blocked) in the Cn*arg1*Δ mutant. The secretome profile was also altered in the Cn*arg1*Δ mutant and this may reflect an altered cell wall proteome and the unusual cell wall architecture. The N-linked mannosylation defect was also absent in the Cn*kcs1*Δ mutant. The Cn*kcs1*Δ mutant was poorly recognized by phagocytes. This is in contrast to the Cn*arg1*Δ mutant which was readily phagocytosed. Both mutants were compromised in utilizing carbon sources other than glucose. This metabolic defect is predicted to impact negatively on their ability to survive in the low glucose environment of the host lung (Garnett et al., 2012). Both mutants were avirulent in a mouse inhalation model (no mice exhibited symptoms of infection up to 60 days post-infection). However, Cn*arg1*Δ infection was cleared rapidly while *kcs1*Δ infection resulted in lower lung burdens and no dissemination to the CNS. These results suggested that IP_7_ is an important metabolite in promoting fungal fitness and dissemination. Reduced recognition of Cn*kcs1*Δ cells by primary or immortalized monocytes and failure to elicit a strong immune response *in vivo* and *in vitro* correlated with reduced exposure of mannoproteins on the Cn*kcs1*Δ cell surface.

Taken together it was concluded that IP_7_ is the most crucial of the IPs for cryptococcal cellular function, fitness and virulence, being essential for fungal metabolic adaptation to the host environment, immunorecognition, dissemination, and pathogenicity (Lev et al., 2015). Functions for IP_7_ have also been identified in *S. cerevisiae*, and include the promotion of resistance to salt stress, cell wall integrity and vacuolar morphogenesis (Dubois et al., 2002) and autophagy (Taylor et al., 2012).

RNAseq analysis of the IPK deletion mutant set in *C. neoformans* (*arg1*Δ*, ipk1*Δ, and *kcs1*Δ) supports a role for IPs in the regulation of gene expression, with genes involved in mRNA translation and ribosomal biosynthesis being more highly expressed than in the WT strain (Lev et al., 2015). The RNAseq data also showed that the pattern of disregulated gene expression is mostly similar in the 3 CnIPK mutants (Li et al., 2016). Since IP_7_ is commonly absent in the 3 IPK mutants, the changes can therefore be attributed predominantly to the absence of IP_7_. IP_7_ may modulate gene expression via interaction with transcription and translation machinery or via pyrophosphorylation of its components as discussed below. Alternatively, altered gene expression in the absence of IP_7_ could be due to compensatory changes.

As mentioned above for IP_3_ kinases, IPKs can perform functions independent of their catalytic activity, such as acting as scaffolds to tether proteins into complexes as has been described for ScArg82 (see above). They also have roles in subcellular protein targeting and DNA binding (Rauch et al., 2011). To investigate whether this is true of CnKcs1, a “kinase-dead” mutant was created by altering critical catalytic residues using site-directed mutagenesis, and its phenotype compared with that of Cn*kcs1*Δ. The same attenuation in phenotype was observed in both mutants i.e., a cell wall integrity and high temperature growth defect, reduced *LAC1* expression, laccase activity and melanin production and larger and more mucoid capsules (Lev et al., 2015) (summarized in Figure 2). The phenotypes observed for both the Cn*kcs1*Δ mutant and the kinase-dead mutant are therefore due solely to the absence of its enzymatic product IP_7_ as IP_8_ does not contribute to the virulence profile.

Kcs1 (presumably via IP_7_) also plays a role in inositol homeostasis in *S. cerevisiae* and *C. neoformans* (Ye et al., 2013; Liao et al., 2018). By comparing the phenotype of WT and the *kcs1*Δ mutants, Kcs1 was shown to negatively regulate inositol uptake and catabolism. However, in contrast to Kcs1 function in *S. cerevisiae*, Kcs1 did not regulate inositol biosynthesis in *C. neoformans*. Irrespective of the presence of glucose, inositol also repressed *KCS1* gene expression in *C. neoformans*, potentially impacting PP-IP_5_/IP_7_ levels (El Alami et al., 2003).

### Role of IP_6_ and 5-PP-IP_4_

IP_6_ is the most abundant IP species. However, by comparing the phenotypes of Cn*ipk1*Δ, Cn*ipk1*Δ*kcs1*Δ, and Cn*kcs1*Δ, IP_6_ was shown to not play a significant role in cryptococcal virulence. This is presumably because other IP and PP-IP species can compensate for its absence, or for the absence of IP_7_ as discussed below. Thus, targeting IP_6_ production may not be the best approach for drug development.

Although Kcs1 is the major IP_6_ kinase in *C. neoformans*, phosphorylating IP_6_ to create IP_7_, it also acts as an IP_5_ kinase when IP_6_ is absent (i.e., in the Cn*ipk1*Δ mutant). The IP_5_ that accumulates in Cn*ipk1*Δ is converted by CnKcs1 to another pyrophosphate species, 5-PP-IP_4_, via the addition of a phosphoryl group to the existing phosphate at position five (see Figure 1) (Li et al., 2016). 5-PP-IP_4_ is therefore only detected when *IPK1* function is disrupted and its physiological relevance remains to be determined. Relative to IP_7_, 5-PP-IP_4_ is produced by CnKcs1 in significant quantities when CnIpk1 function is abolished, and the presence of 5-PP-IP_4_ in Cn*ipk1*Δ correlates with an increased virulence phenotype relative to that of Cn*ipk1*Δ*kcs1*Δ and Cn*kcs1*Δ (Li et al., 2016). This suggests that the “bypass pathway” responsible for production of PP-IP_4_ can partially compensate for the absence of IP_7_ (and hence its progenitor IP_6_) in the Cn*ipk1*Δ mutant. This functional redundancy is most likely due to the structural similarity between the two PP-IP species (see Figure 1). Interestingly, unlike Cn*ipk1*Δ*kcs1*Δ and Cn*kcs1*Δ, Cn*ipk1*Δ lung and brain burdens were high and resulted in a 20% mortality rate by 50 days post infection (Li et al., 2016). The Cn*ipk1*Δ mutant is therefore well-tolerated in the mouse and slow to be detected by the host immune system. Elevated 5-PP-IP_4_ in Cn*ipk1*Δ correlated with the rescue of alternative carbon source utilization but not with the rescue of laccase and urease production and ability to grow under oxidative/ nitrosative stress (Li et al., 2016).

In summary, a comparison of the phenotypes of CnIPK mutants confirmed that CnArg1 and IP_7_ are the most crucial IPK and IP species, respectively, for fungal cellular function and virulence, and allowed IP_7_-dependent and IP_7_-independent functions of CnArg1 to be distinguished (Figure 2). Possible reasons for IP_7_-deficent Cn*arg1*Δ having the most attenuated virulence phenotype of all of the IPK mutants, are the loss of IP_4/5_ and/or elevated levels of PIP_2_ and IP_3_. The potential impact of these changes in the IP profile is discussed below.

## IP_7_ and the Regulation of Phosphate Homeostasis in Fungi

The pleiotropic phenotype of the IP_7_-deficient Cn*kcs1*Δ strain confirms that many of the important functions of CnArg1 are conveyed via IP_7_ (Lev et al., 2015). It was demonstrated that both the ability to synthesize IP_7_ and to activate the fungal phosphate acquisition (PHO) pathway, are essential for *C. neoformans* to grow in the blood and disseminate from the lung to the brain (Lev et al., 2015, 2017). This is consistent with the IPK and PHO pathways being connected in *C. neoformans* via IP_7_, as has been reported in *S. cerevisae* and *S. pombe*. No information is available on a possible connection between IP_7_ and the PHO pathway in other pathogenic fungi.

IP_7_ is extraordinarily rich in phosphate and its biosynthesis is directly tied to phosphate availability. Moreover, it was suggested that in *S. cerevisiae*, IP_7_ serves as a cellular energy sensor due to its high energy pyrophosphate (PP) bond. The K_m_ of ScKcs1 for ATP is 1.1–1.4 mM, which is similar to the physiological concentration of ATP in the absence of nutritional stress (Wilson et al., 2013). Thus, when ATP is depleted, IP_7_ should also be depleted.

### How the PHO Pathway Becomes Activated

The fungal PHO pathway is activated when phosphate becomes scarce in the environment or at alkaline pH. The cyclin-dependent kinase inhibitor Pho81, then inhibits the cyclin-dependent kinase Pho85, which ceases phosphorylation of the transcription factor Pho4. The helix-loop-helix transcription factor Pho4, is exported from the nucleus when it is phosphorylated, and imported into the nucleus when it is dephosphorylated. In *S. cerevisiae* and *C. albicans*, Pho4 associates with Pho2 to trigger expression of genes involved in phosphate acquisition. In *C. neoformans*, Pho4 is the sole transcription factor regulating PHO gene expression. One of the PHO gene products, secreted acid phosphatase, is widely used to monitor PHO pathway activation in variety of fungal species using a simple and robust colorimetric enzyme assay. The PHO pathway was recently implicated in cryptococcal pathogenesis following the demonstration that CnPho4, and therefore the expression of CnPho4-dependent genes, is essential for growth of *C. neoformans* in the blood and for cryptococcal dissemination to the brain (Lev et al., 2017). Ikeh et al also showed that CaPho4 is essential for disseminated candidiasis (Ikeh et al., 2016).

A number of earlier studies in *S. cerevisiae* reported perturbation of PHO gene expression in ScIPK mutants: Auesukaree et al (Auesukaree et al., 2005) demonstrated that the acid phosphatase encoding gene, *PHO5*, was constitutively expressed in the Sc*plc1*Δ, Sc*arg82*Δ, and Sc*kcs1*Δ mutants and therefore not responsive to phosphate deprivation. Similarly, El Alami reported up-regulation of *PHO5*, and other PHO genes, in Sc*arg82*Δ and Sc*kcs1*Δ under non-inducing (phosphate replete) conditions (El Alami et al., 2003). There is some controversy as to which IP_7_ isoform is involved in PHO pathway activation: Lee et al demonstrated that ScVip1-derived IP_7_ (the 1-PP-IP_5_ isoform) interacts non-covalently with ScPho81 in the context of the ScPho80-Pho85-Pho81 complex, thus preventing Pho4 from accessing the kinase active site (Lee et al., 2007, 2008). Their data showed a corresponding increase in IP_7_ following phosphate deprivation. However, these findings are controversial: firstly, the level of ATP declines as a result of phosphate deprivation, rendering an increase in ATP-dependent IP_7_ production unlikely (Choi et al., 2017). In fact, Lonetti et al demonstrated a decrease in the abundance of ScKcs1-derived IP_7_ (5-PP-IP_5_) under similar conditions (Lonetti et al., 2011). It is possible that due to the low affinity of ScKcs1 for ATP, the activity of ScKcs1 is highly sensitive to ATP fluctuations, while the activity of ScVip1 is not affected, leading to an observed increase in IP_7_. Secondly, in a study assessing PHO pathway activation in IP6K mutants, it was shown that in the absence of ScKcs1, but not ScVip1, expression of *PHO5* and *PHO84* is constitutive, implicating ScKcs1-derived 5-PP-IP_5_, not Vip1-derived 1-PP-IP_5_, in PHO pathway regulation (Nishizawa et al., 2008; Choi et al., 2017).

### IP_7_ Interacts With SPX Domains of PHO Pathway Components

SPX domains are found in eukaryotic proteins involved in phosphate homeostasis. In a recent breakthrough study, Wild et al demonstrated that SPX domains in *S. cerevisiae* and the model plant, Arabidopsis, provide a positively-charged binding module for various PP-IPs (Wild et al., 2016). They found that SPX domains bind IP_6_ and IP_7_ with high affinity (K_d_ in nM range) and other IPs including IP_3_/IP_4_/IP_5_ and free orthophosphate, with markedly lower affinity (K_d_ in μM range). They hypothesized that the abundance of IP_7_, which changes in response to phosphate deprivation, communicates cellular phosphate levels to SPX domain proteins to trigger an adaptive response (Wild et al., 2016). Whether IP_7_ interacts with SPX domains of PHO pathway components in fungal pathogens to fine-tune phosphate homeostasis, remains to be investigated.

The genome of *S. cerevisiae* encodes 10 SPX domain proteins, all containing the SPX domain at the N-terminus [(Desfougeres et al., 2016), reviewed in Azevedo and Saiardi (2017)]. Four of these proteins are components of the vacuolar transport complex (VTC). The others are Pho91, Pho87, Pho90, Gde1/Pho81, Syg1, and Xpr1. SPX is derived from Syg1, Pho81, and Xpr1, which are the proteins in which the SPX domain was first discovered. The VTC complex couples the production of inorganic polymers of phosphate (polyphosphates or polyP) with their transport to vacuoles and acidocalciosome-like vacuoles where they are stored. PolyP in these stores are promptly mobilized as a source of phosphate when the intracellular phosphate concentration is insufficient. IP_6_ and 5-PP-IP_5_ were shown to bind to the SPX domains of the VTC protein complex, stimulating polyP biosynthesis in isolated yeast vacuoles *in vitro* (Wild et al., 2016). Interestingly, IP_6_ and IP_7_ (the 5-PP-IP_5_ isoform) also bind to the SPX domain in the Na^+^/P_i_ symporter Pho91, triggering phosphate and sodium release from yeast vacuoles *in vitro* (Potapenko et al., 2018). Pho87 and Pho90 are low affinity plasma membrane phosphate transporters, which function when phosphate is available. In these proteins, the SPX domain performs an inhibitory function, limiting low-affinity phosphate uptake (Hurlimann et al., 2009). Pho81/GDE1 is the CDK inhibitor of the PHO pathway but also has glycerophosphodiesterase activity, which is thought to generate glycerol-phosphate during phosphate starvation (Fisher et al., 2005). Syg1 is predicted to be a phosphate transporter based on its similarity to the mammalian phosphate exporter Xpr1 (Giovannini et al., 2013). PP-IP interactions with the SPX domain of Syg1 have not been investigated. Of the SPX domain proteins in *S. cerevisiae*, only four have homologs in *C. neoformans*, including CnPho91, CnPho81, CnSyg1, and the CnVtc4 component of the VTC complex. Vtc4 and Pho81 have a similar function in *C. neoformans* and *S. cerevisiae* (Kretschmer et al., 2014; Toh-e et al., 2015), while the roles for CnPho91 and CnSyg1 have not been determined.

In summary, identification of SPX domain proteins as cellular IP_7_ receptors is a milestone in our understanding of how these molecules orchestrate numerous cellular functions. Given the different functions of each SPX domain protein, the impact of each SPX-IP_7_ interaction in fungal pathogenicity should be assessed on a case by case basis to gain a full understanding of this novel regulatory mechanism.

## PP-IPs and the Regulation of Stress Response

In *S. cerevisiae*, all PP-IP species regulate chromatin remodeling, working in parallel with the target of rapamycin complex 1 (TORC1) pathway to control class I histone deacetylase Rpd3L activity and elicit global transcriptional changes in response to stress or starvation (Worley et al., 2013). Phenotypic cluster analysis of the cryptococcal kinome (Lee et al., 2016) identified the IP_5_ kinase Ipk1, and therefore potentially its distal PP-IP products, as working in the same pathway as the essential TORC1 kinase complex, rather than working in parallel as reported by Worley et al. (2013). In support of this, phosphate was identified as a nutrient monitored by TOR in *Candida albicans* (Liu et al., 2017). PP-IPs have also been reported to regulate apoptosis and telomere length by antagonizing the actions of PI3K-related protein kinases in the nucleus (Saiardi et al., 2005).

## PP-IPs Exert Regulatory Effects by Pyrophosphorylation

Direct binding of IP_7_ to SPX proteins as described above, is only one mechanism by which PP-IPs exert pleiotropic effects in eukaryotic cells. However, PP-IPs are unique in that they can also modulate the activity of their target proteins by pyrophosphorylating them. This non-enzymatic reaction involves transferring the terminal phosphoryl group from their PP moiety to a pre-existing phosphate on the target, creating a new PP bond (Bhandari et al., 2007). For example, IP_7_ regulates yeast rRNA synthesis (a first step in ribosome biosynthesis) via pyrophosphorylation of RNA polymerase I (Thota et al., 2015). A recent study in mammalian cells by Chanduri et al demonstrated that IP_7_ regulates dynein-driven cytoplasmic vesicular transport along the microtubule. Ser51 in the dynein intermediate chain is pyrophosphorylated by IP_7_, and this modification promotes the interaction of dynein with the p150^Glued^ subunit of dynactin, which recruits the motor to vesicles. This mechanism provides an explanation for how PP-IPs affect intracellular vesicular trafficking in mammalian cells (Chanduri et al., 2016; Saiardi, 2016). In a high throughput study in *S. cerevisiae*, multiple potential pyrophosphorylation targets (as well as IP_6_ -and IP_7_-interacting proteins) were identified using specially synthesized resins. These resins expose the non-hydrolysable IP_7_ analog, 5-PCP-IP_5_ (or IP_6_) to capture interacting proteins from whole yeast cell lysates (Wu et al., 2016). These studies have paved the way for investigating the consequences of such interactions in promoting fungal pathogenesis.

## Mechanistic Insight into the Role of IP_7_ Precursors in Fungal Cellular Function and Pathogenicity

In fungal pathogens, it is still not clear whether IP_3/4/5_ serve predominantly as precursors for the synthesis of IP_7_, whether these metabolic precursors themselves modulate cellular function, and if so how? Evidence from fungal and animal model organisms suggests that IP_3/4/5_ evolved to have independent roles in cellular function. These studies provide a rich source of information for continuing investigation into the mechanisms by which these IP species exert their roles in fungal pathogenicity.

As previously mentioned, the metabolic profile of the Cn*arg1*Δ mutant is characterized by the absence of IP_4−6_ and PP-IP_5_ and the accumulation of IP_3_ and PIP_2_. In *S. cerevisiae* accumulation of PIP_2_ is responsible for defects in organization of actin cytoskeleton, cell wall, vacuolar morphology, endocytosis, and clathrin-mediated sorting between the Golgi and endosomes (Stefan et al., 2002). IP_3_ accumulation could also impact negatively on cellular function in the Cn*arg1*Δ mutant. For example, elevated IP_3_ could compete with PIP_2_ for the binding of cytoplasmic proteins with a pleckstrin homology (PH) domain (Varnai et al., 2002). Elevated IP_3_ could also potentially alter calcium homeostasis in *C. neoformans*, which has implications for virulence as altering calcium homeostasis by ablating the function of cryptococcal vacuolar calcium channels attenuates virulence in animal models (Squizani et al., 2018). In *C. neoformans*, the source of IP_3_ is PIP_2_, which is hydrolysed by PLC1 (Lev et al., 2013). In mammalian cells, PLC1-derived IP_3_ causes the release of Ca^2+^ from the endoplasmic reticulum (ER), where calcium is predominantly stored, following engagement of an IP_3_ receptor (Mikoshiba et al., 1994; Bergsma et al., 2001). This leads to activation of the serine/threonine phosphatase, calcineurin. However, the spike in IP_3_ in the Cn*arg1*Δ mutant did not lead to calcineurin activation in *C. neoformans* (Lev et al., 2013). Despite this observation, whether or not elevated IP_3_ has an impact on intracellular calcium levels in Cn*arg1*Δ, and the subcellular location of any calcium spikes, remains to be determined.

In *C. albicans* it was shown that suppressing *IPK2* expression, which is predicted to cause IP_3_ accumulation, did in fact correlate with a spike in cytoplasmic calcium (Li J. et al., 2017). This is consistent with IP_3_ playing a role in regulating calcium homeostasis. The authors concluded that elevation in cytosolic calcium was due to calcium release from the vacuole due to IP_3_-mediated activation of the vacuolar calcium channel, Yvc1. In contrast to mammalian cells, the major calcium storage organelle in fungi is the vacuole, not the ER. Their results are consistent with those observed in the *S. cerevisiae ARG82/IPK2* deletion mutant, where elevated IP_3_ caused constitutive activation of Yvc1, leading to an increase in cytoplasmic calcium (Tisi et al., 2004; Bouillet et al., 2012). Whether Yvc1 is a receptor for Arg1/Ipk2-derived IP_3_ in *C. albicans* and *C. neoformans*, remains to be determined.

In mammalian cells inositol 1,4,5,6-tetrakisphosphate (IP_4_) acts as intermolecular glue to facilitate binding of class 1 histone deacetylases to their cognate co-repressor to form transcription repression complexes. This mechanism of HDAC activation is thought to be conserved in class I HDACs from yeast to humans (Watson et al., 2012, 2016; Millard et al., 2013). A different IP_4_ isoform (inositol 1,3,4,5-tetrakisphosphate), inositol 1,3,4,5,6-pentakisphosphate (IP_5_), and inositol hexakisphosphate (IP_6_) have been reported to control protein phosphorylation by regulating the activity of the serine/threonine kinase casein kinase 2 (CK2), which phosphorylates a diverse array of proteins and is thought to be responsible for 20% of the eukaryotic phosphoproteome (Solyakov et al., 2004).

Numerous studies report a direct binding role for IP_6_ in the regulation of nuclear processes in eukaryotes. While IP_6_ does not have a dominant role in cryptococcal pathogenesis, it has been shown to bind to the mRNA export factor, Gle1, in *S. cerevisiae* to regulate mRNA export (Alcazar-Roman et al., 2010) and to the RNA editing enzyme, ADAR2, in humans to promote its stability and activity (Macbeth et al., 2005). IP_6_ binding also has a role in signaling, regulating oligomerization and cellular localization of Arrestin-2 (Milano et al., 2006). More recently, human host-derived IP_6_ has been reported to play a role in HIV-1 assembly and maturation (Dick et al., 2018).

## Focus of Future Studies in Determining the Roles of IPs and PP-IPs in Fungal Pathogenicity

Future work in fungal pathogenesis needs to address whether, and which, transcription factors respond to IPs, and how IPs regulate virulence-related signaling pathways, including the PHO pathway. Crosstalk between IPs and Ras signaling has been reported in the basidiomycete, *Schizophyllum commune*, where lithium-induced activation of the IPK pathway, leading to increased levels of PP-IPs, was not observed in a Ras1 mutant (Murry et al., 2019).

## IPKs as Novel Antifungal Drug Targets

The health and socioeconomic costs associated with the treatment of invasive fungal disease are enormous with estimates of ~2.6 billion per year in the US alone (Benedict et al., 2019). Reasons for this include the availability of only a small arsenal of antifungals with few targets. Some of the drawbacks of these drugs include poor bioavailability, lack of broad-spectrum efficacy, and toxicity. Furthermore, drug resistance is emerging. The gold standard for treating cryptococcal meningoencephalitis is a combination of the polyene amphotericin B and the uracil derivative 5-fluoro-uracil. 5-fluoro-uracil is not suitable for monotherapy because of the rapid development of resistance and hematologic toxicities. Similarly, amphotericin B is nephrotoxic and requires intravenous administration. Fluconazole is fungistatic and most commonly used for maintenance (suppressive) therapy and as an alternative to amphotericin B, especially in resource-limited regions (Sloan et al., 2009; Perfect et al., 2010). Although fluconazole inhibits the growth of *Cryptococcus* at low drug concentrations, it predisposes the patient to clinical relapse and the development of drug resistance (Sloan et al., 2009; Krysan, 2015). Echinocandins are the newest class of antifungal drug introduced into clinical practice in the early 2000s (Roemer and Krysan, 2014). Unfortunately, the echinocandins are not effective against *Cryptococcus* (Perfect et al., 2010). Given the limitations of our current antifungal drug arsenal, targeting essential pathways other than ergosterol and cell wall biosynthesis would be advantageous. The IPK pathway represents one such target particularly because it has a pleotropic role in virulence affecting the production of numerous virulence factors and is essential for promoting disseminated meningitis which is fatal without treatment. In the case of targeting IPKs, existing kinase inhibitors could be repurposed or entirely new inhibitors could be developed.

Another advantage of targeting the fungal IPK pathway is that there are no signaling redundancies. The pathway is linear and therefore non-redundant in fungi, while in humans there is a high degree of redundancy particularly in the steps converting IP_3_ to IP_5_. Thus, the chances of fungal IPK inhibitors blocking conversion of IP_3_ to IP_5_ in the host is minimal.

Several candidate molecules have been reported to inhibit IPKs, but not protein kinases, with different degrees of selectivity. The cell permeable purine-based compound, N2-(m-(trifluoromethyl)benzyl) N6-(p-nitrobenzyl) purine (TNP), selectively inhibits both human IP3K and IP6K enzymes by competing with ATP (Chang et al., 2002; Padmanabhan et al., 2009). The selectivity of TNP for IP6K is significantly higher than for IP3K. Similar to the effects of *KCS1* deletion, TNP causes vacuolar fragmentation in *S. cerevisiae* (Padmanabhan et al., 2009). In addition to TNP, a number of natural and synthetic polyphenolic compounds were found to inhibit all three mammalian IP3K isoforms, as well as IPMK. One of these compounds, ellagic acid, is found in fruits and vegetables and has antiproliferative and antioxidant properties (Khanduja et al., 1999; Seeram et al., 2005). Chlorogenic acid, a major polyphenolic compound in coffee, selectively inhibited human IPMK at IC_50_ of 1.15 μM (Mayr et al., 2005). Chlorogenic acid, like a number of other natural polyphenols, was reported to suppress the growth of tumor cells *in vitro* (Feng et al., 2005; Yamagata et al., 2018). Furthermore, derivatives of chlorogenic acid exhibited antifungal activity against *C. neoformans, C. albicans*, and *A. fumigatus* (Ma et al., 2007). Polyphenolic compounds inhibit enzymes other than IPKs, in particular Topoisomerase II, but in many cases the IC_50_ for other enzymes is much higher than the IC_50_ for IPKs. Inhibition of IP3K and IPMK by polyphenolics is of a linear mixed type with respect to ATP binding, and of a linear non-competitive type with respect to IP_3_. This complex mechanism of binding is considered advantageous for developing highly selective inhibitors (Mayr et al., 2005). Thus, chemical derivatisation of polyphenolic compounds represents a promising strategy for developing novel antifungal molecules.

Human protein and lipid kinases have been widely investigated as targets for specific inhibitors as they are directly involved in a variety of disease-associated processes, especially cancerous growth. The key challenge is designing kinase inhibitors with the desired target specificity. The ideal inhibitors should be selective only for their target kinase (have minimal off target effects) and potent (only require a low dose) to reduce the potential for liver and kidney toxicity. However, this is usually almost impossible to achieve given that the human kinome is comprised of over 500 protein kinases. Despite this difficulty, as of 2014, 30 kinase inhibitors were approved for clinical use, mainly for oncological treatment purposes (Fabbro, 2015). The majority of these inhibitors target the ATP binding site. Conservation of the ATP binding site often causes inhibitors to cross-react with other kinases resulting in compounds with promiscuous profiles. Inhibitor specificity can be gained by designing inhibitors that bind to two different sites, one of which is the ATP-binding site. Alternatively, some inhibitors use structural features of the target kinase that do not include the ATP binding pocket (Fabbro, 2015). The methods used for developing human kinase inhibitors and the collections of compounds already available, provide opportunities for repurposing as well as creating new inhibitors with desired selectivity for fungal IPKs. Obtaining crystal structures for fungal IPKs will also facilitate drug discovery. So far the only IPK from a fungal pathogen that has been crystalized is Ipk1 (IP_5_ kinase) from *C. neoformans* (Oh et al., 2017).

## Conclusions

Multiple studies demonstrate that IPs and PP-IPs play major roles in cellular function in all eukaryotes, from humans to fungi. *C. neoformans* has provided an essential model for elucidating the IPK pathway in fungal pathogens, and for demonstrating that IPKs play crucial roles in growth, fitness and virulence, predominantly via the more distal product IP_7_. *In silico* analysis of fungal genomes demonstrate that this pathway is also present in the major human fungal pathogens, *C. albicans* and *A. fumigatus*. Although inability to completely ablate IP_3_ kinase function in *C. albicans* has hindered progress in determining the impact of IPK function on the pathogenicity of *C. albicans* in animal infection models, the difficulties experienced point to this kinase having an essential role in fungal fitness. Furthermore, due to technical difficulties in detecting and quantifying IPs and PP-IPs, the mechanism of action of these metabolic messengers is only partially understood and efforts to study this in fungal and mammalian cells is ongoing. Despite the ubiquity of IPs and PP-IPs in nature, the IPK pathways that lead to their synthesis have become more complex and redundant in higher eukaryotes, while the pathway in fungi has remained linear. The lack of redundancy and low sequence conservation, coupled with what is now known about the importance of IPKs in cryptococcal pathogenicity, renders fungal IPKs, particularly the lead kinase Arg1, attractive targets for the development of an anticryptococcal drug. Pending further investigation these drugs could potentially have a broader antifungal specificity.

## Author Contributions

JD and SL wrote the manuscript. CL, TS, AS, and DD proofread the manuscript and provided helpful suggestions.

### Conflict of Interest Statement

The authors declare that the research was conducted in the absence of any commercial or financial relationships that could be construed as a potential conflict of interest.
